# Supplementary material for: The Discovery of Small Allosteric and Active Site Inhibitors of the SARS-CoV-2 Main Protease via Structure-Based Virtual Screening and Biological Evaluation
Source: Molecules. 2022 Oct 9;27(19):6710. doi: 10.3390/molecules27196710 (PMC9572942; doi:10.3390/molecules27196710)
Supplement: Supplementary file 1 [file molecules-27-06710-s001.zip › molecules-1919371-supplementary.pdf]

Supporting Information

The Discovery of Small Leadlike Inhibitors of the SARS-CoV-2 Main Protease via Structure-Based Virtual Screening and Biological Evaluation

Radwa Mahgoub [1,2], Fedah E. Mohamed [3], Lara Alzyoud [1,2], Bassam R. Ali [3,4], Juliana Ferreira [5], Wael M. Rabeh [5], Shaikha S. AlNeyadi [6], Noor Atatreh [1,2], Mohammad A. Ghattas [1,2]*

[1] College of Pharmacy, Al Ain University, Abu Dhabi 64141, United Arab Emirates
[2] AAU Health and Biomedical Research Center, Al Ain University, Abu Dhabi 64141, United Arab Emirates
[3] Department of Genetics and Genomics, College of Medicine and Health Sciences, United Arab Emirates University, Al-Ain 15551, United Arab Emirates
[4] Zayed Centre for Health Sciences, United Arab Emirates University, Al-Ain 15551, United Arab Emirates
[5] Science Division, New York University Abu Dhabi, Abu Dhabi 129188, United Arab Emirates
[6] Department of Chemistry, College of Science, United Arab Emirates University, Al-Ain 15551, United Arab Emirates
* Correspondence: mohammad.ghattas@aau.ac.ae; Tel.: +971-26133275

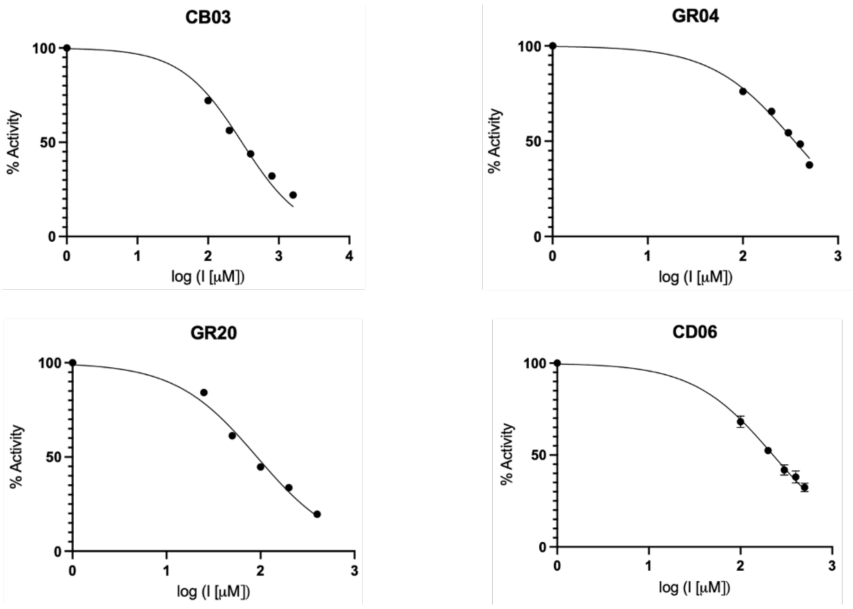

Figure S1. IC_{50} Plots for Top Hits
